# The vocabulary of microbiome research: a proposal

**DOI:** 10.1186/s40168-015-0094-5

**Published:** 2015-07-30

**Authors:** Julian R. Marchesi, Jacques Ravel

**Affiliations:** Cardiff School of Biosciences, Division of Microbiology, Cardiff University, Museum Avenue, Cardiff, CF10 3AT United Kingdom; Centre for Digestive and Gut Health, Imperial College London, Exhibition Road, London, SW7 2AZ United Kingdom; Institute for Genome Sciences, University of Maryland School of Medicine, 801 West Baltimore Street, Baltimore, MD 21201 USA; Department of Microbiology and Immunology, University of Maryland School of Medicine, 685 West Baltimore Street, Baltimore, MD 21201 USA

## Abstract

The advancement of DNA/RNA, proteins, and metabolite analytical platforms, combined with increased computing technologies, has transformed the field of microbial community analysis. This transformation is evident by the exponential increase in the number of publications describing the composition and structure, and sometimes function, of the microbial communities inhabiting the human body. This rapid evolution of the field has been accompanied by confusion in the vocabulary used to describe different aspects of these communities and their environments. The misuse of terms such as microbiome, microbiota, metabolomic, and metagenome and metagenomics among others has contributed to misunderstanding of many study results by the scientific community and the general public alike. A few review articles have previously defined those terms, but mainly as sidebars, and no clear definitions or use cases have been published. In this editorial, we aim to propose clear definitions of each of these terms, which we would implore scientists in the field to adopt and perfect.

## Microbiota

The assemblage of microorganisms present in a defined environment. The term microbiota was first defined by Lederberg and McCray [1] who emphasized the importance of microorganisms inhabiting the human body in health and disease. This microbial census is established using molecular methods relying predominantly on the analysis of 16S rRNA genes, 18S rRNA genes, or other marker genes and genomic regions, amplified and sequenced from given biological samples. Taxonomic assignments are performed using a variety of tools that assign each sequence to a microbial taxon (bacteria, archaea, or lower eukaryotes) at different taxonomic levels from phylum to species.

### Metataxonomics

Metataxonomics is a term we propose and define as the high-throughput process used to characterize the entire microbiota and create a metataxonomic tree, which shows the relationships between all sequences obtained. While viruses are an integral part of the microbiota, no universal viral marker genes are available to perform such taxonomic assignments.

### Metagenome

The collection of genomes and genes from the members of a microbiota. This collection is obtained through shotgun sequencing of DNA extracted from a sample (metagenomics) followed by assembly or mapping to a reference database followed by annotation. Metataxonomic analysis, because it relies on the amplification and sequencing of taxonomic marker genes, is not metagenomics. Metagenomics is the process used to characterize the metagenome, from which information on the potential function of the microbiota can be gained.

Metagenomics was first used by Handelsman et al. [2]; however, it was in the context of what the authors called functional metagenomics, an approach where random fragments of environmental DNA are cloned into a suitable vector for maintenance in a surrogate host for functional screening, looking for gain of function in the surrogate host.

### Microbiome

This term refers to the entire habitat, including the microorganisms (bacteria, archaea, lower and higher eurkaryotes, and viruses), their genomes (i.e., genes), and the surrounding environmental conditions. This definition is based on that of “biome,” the biotic and abiotic factors of given environments. Others in the field limit the definition of microbiome to the collection of genes and genomes of members of a microbiota. It is argued that this is the definition of metagenome, which combined with the environment constitutes the microbiome. The microbiome is characterized by the application of one or combinations of metagenomics, metabonomics, metatranscriptomics, and metaproteomics combined with clinical or environmental metadata.

### Metabolomics

This term describes the analytical approaches used to determine the metabolite profile(s) in any given strain or single tissue. The resulting census of all metabolites present in any given strain or single tissue is called the *metabolome*. Most commonly used platforms to characterize the metabolome include nuclear magnetic resonance (NMR) spectroscopy and mass spectrometry (MS) linked to a liquid chromatography separation system.

### Metabonomics

The term is a variant of the metabolomic approach; however, it describes the approach used to generate a metabolite profile(s) from complex systems, e.g., mammals in which more than one strain or tissue has contributed to the total metabolite pool, for example, fecal water, urine, or plasma. This term avoids the clumsy use of meta-metabolomics and was first defined by Jeremy Nicholson [3].

### Metatranscriptomics

This term refers to the analysis of the suite of expressed RNAs (meta-RNAs) by high-throughput sequencing of the corresponding meta-cDNAs. This approach provides information on the regulation and expression profiles of complex microbiomes.

### Metaproteomics

First coined by Rodriguez-Valera [4] and refined by Wilmes and Bond [5], this term refers to the large-scale characterization of the entire protein complement of environmental or clinical samples at a given point in time. The method indiscriminately identifies proteins from the microbiota and the host/environments (metagenome). Computational analyses afford assignments of these proteins to their biological origins. It is often performed using liquid-chromatography-based separation coupled to mass spectrometry for peptide identification.

### Misnomers and correct usage of the terms

Misnomers are often found in studies discussing metataxonomic analyses relying on sequencing and analysis of 16S rRNA genes. In the literature, one can find the use of “16S survey,” “16S sequencing,” or “16S analysis,” for example. There is no such thing as “16S.” The “S” in 16S is a non-SI unit for sedimentation rate and stands for the Svedberg unit. The Svedberg unit offers a measure of particle size based on its rate of travel in a tube subjected to high *g* force. The small subunits of the bacterial and archaeal ribosomes are 30S and comprise one structural 16S ribosomal RNA (rRNA, ~1540 nucleotides) bound to 21 proteins. Thus, we would like to argue that the proper terms should be “16S rRNA genes” or “16S rRNA gene sequencing/analysis.”

Additionally, the word microflora has been used for a long time in the scientific and medical literature. However, its definition does not justify its use to describe microbial communities associated with human (i.e., microbiota). Its definition has evolved over time, but remains “microscopic plants, or the plants or flora of a microhabitat.” The origin of the definition dates back to the early 1900s. Furthermore, the definition of the word “flora” further highlights the inappropriateness of the word microflora in the microbiome scientific literature: “the plants of a particular region or period, listed by species and considered as a whole” or “a work systematically describing plants” or “plants, as distinguished from fauna.” The definition of flora dates back to mid 1600s and has its origin in the Latin name “Flora,” the Roman goddess of flowers and the Latin word “flor,” meaning flower. These definitions and their origins make it obvious that “microflora” refers to plants and not microbes. While some dictionaries are now including a third definition for microflora, “the aggregate of bacteria, fungi, and other microorganisms normally occurring on or in the bodies of humans and other animals: intestinal flora,” these newly added definitions are the results of over one century of misuse of the word, driven by a limited understanding of the microbes associated with humans. Our knowledge of microbial communities is such that the scientific community should not continue to use the word in the scientific literature. It is time to change, and we suggest that to describe the assemblage of microbes living in a microhabitat we use “microbiota.” Interestingly, microflora is almost exclusively used in the literature referring to microbial community associated with human or animal, but rarely in those associated with the environment. We believe that microflora has still its place in the popular literature or in a yogurt/probiotic advertisement destined to the general public, but it does not in the scientific and medical literature.

The public, the scientific popular press, medical doctors, and other scientists need to be educated, but this will come if the scientific community adopts a common language. The word microbiota is adequate and appropriate to describe the composition and abundance of microbial communities whether they inhabit the human body or the environment.

This editorial was informed from papers and other communications we have had with colleagues. We hope that a consensus use of these terms could be adopted in the near future. This editorial aims at stimulating a discussion and standardizing the vocabulary of microbiome research. *Microbiome* will continue to strive toward a standardization of the vocabulary used in this ever-expanding field of research.
